# Seroprevalence of porcine proliferative enteropathy among wild boars in the Republic of Korea

**DOI:** 10.1186/1746-6148-10-5

**Published:** 2014-01-06

**Authors:** Jung-Yong Yeh

**Affiliations:** 1 Division of Life Sciences, Incheon National University, 406-772, Academy-ro 119, Yeonsu-gu, Incheon, Republic of Korea

**Keywords:** *Lawsonia intracellularis*, Serology, Wild boar

## Abstract

**Background:**

The importance of the wild boar as a reservoir of *Lawsonia intracellularis* was assessed by investigating the seroprevalence of this pathogen among wild boars in the Republic of Korea. The extent of exposure to *L. intracellularis* among wild boars (*Sus scrofa coreanus*) was monitored by a country-wide serological survey using an immunoperoxidase monolayer assay.

**Results:**

In this study, antibodies to *L. intracellularis* were observed in 165 of 716 clinically healthy wild boars tested. The overall apparent prevalence calculated directly from the sample and the true prevalence calculated based on the accuracy of the test method were 23.0% (95% confidence interval: 20.0-26.3%) and 25.6% (95% confidence interval: 23.9-27.2%), respectively. Serologically positive animals were found in all the tested provinces.

**Conclusions:**

Our results confirm that *L. intracellularis* is present in the wild boar population worldwide, even in Far East Asia. Despite the high seroprevalence shown in wild boars, further studies are warranted to evaluate their potential as a reservoir species because seroprevalence does not prove ongoing infection nor shedding of the bacteria in amounts sufficient to infect other animals. It should also be determined whether the wild boar, like the domestic pig, is a natural host of *L. intracellularis.*

## Background

*Lawsonia intracellularis* causes porcine proliferative enteropathy (PPE) in domestic pigs (*Sus scrofa domestica*), and today, PPE is present in all swine-producing areas worldwide [1]. PPE has been described as a disease most that is common among grower and finisher pigs [2]. Although pig-to-pig transmission is considered to be the primary route of infection [3], little is known about the mechanisms of transmission of *L. intracellularis* and the epidemiology of PPE, especially in wildlife. Although a few studies on *L. intracellularis* infection in wild boars have been conducted in European countries [4-6], similar studies from Asia are lacking. Tomanova *et al.* reported that *L. intracellularis* was present in wild boars in the Czech Republic, using PCR analysis of intestinal tissues and/or serological examination in 2002 and 2006 [4,5], whereas Jacobson et al. found no evidence of fecal shedding in the Swedish wild boar population [6]. While our manuscript was in preparation, the prevalence of antibodies to *L. intracellularis* in farmed European wild boars was also reported [7]. The aim of the present study was to assess the importance of the wild boar (*Sus scrofa coreanus*) population as a potential reservoir for *L. intracellularis* in the Republic of Korea (ROK). A nationwide prevalence survey was carried out to determine the *L. intracellularis* seroprevalence and we report the serological prevalence of *L. intracellularis* infection in captive wild boars from fields and forests in the ROK.

## Results and discussion

In this study, 716 serum samples collected from clinically healthy wild boars in fields and forests were examined by immunoperoxidase monolayer assay (IPMA). Antibodies to *L. intracellularis* were observed in 165 wild boars. The overall apparent prevalence calculated directly from the sample and the true prevalence calculated based on the accuracy of the test method were 23.0% (95% confidence interval (CI): 20.0-26.3%) and 25.6% (95% CI: 23.9-27.2%), respectively. Table 1 presents the prevalence of antibodies to *L. intracellularis* in the wild boar population from 8 provinces in the ROK. Serologically positive animals were found in all the tested provinces. Gyeonggi province, which produces more domestic pigs than any other province in the ROK [8], and Gangwon province showed high (true) prevalence, at 38.1% (95% CI: 34.7%-41.5%) and 31.2% (95% CI: 27.1-35.3%), respectively.

**Table 1:** **Seroprevalence and 95% confidence interval for *Lawsonia intracellularis *** **in wild boars in the Republic of Korea from 2010 to 2011**

**Locality**	**No. of tested animals**	**No. of positive animals**	**Apparent prevalence (AP)**	**True prevalence (TP)**^**a**^	**Lower 95% confidence limit of TP**	**Upper 95% confidence limit of TP**
Gangwon	128	36	28.1	31.2	27.1	35.3
Gyeonggi^b^	210	72	34.3	38.1	34.7	41.5
Gyeongnam^c^	58	16	27.6	30.7	24.6	36.7
Gyeongbuk^d^	102	12	11.8	13.1	9.8	16.4
Jeonnam^e^	96	8	8.3	9.2	6.3	12.1
Jeonbuk	23	3	13.0	14.4	7.2	21.7
Chungnam^f^	32	6	18.8	20.9	13.8	28.0
Chungbuk	67	12	17.9	19.9	15.1	24.7
Total	716	165	23.0	25.6	23.9	27.2

^a^TP = (AP + specificity-1)/(sensitivity + specificity-1) [9-12]. Specificity and sensitivity of the test employed in this study were according to the previous report [13].

^b^Seoul Metropolis and Incheon Metropolitan City included; ^c^Ulsan and Busan Metropolitan Cities included; ^d^Daegu Metropolitan City included; ^e^Gwangju Metropolitan City included; ^f^Daejeon Metropolitan City included.

Previous studies have shown the presence of *L. intracellularis* among wild boar in Europe [4,5]. The results of the current study show that *L. intracellularis* infections also occur in wild boar populations in Asia. The previously reported prevalence of *L. intracellularis* determined by PCR analysis of intestinal tissues and/or serological examination of wild boars were as follows: 59.2% (95% CI: 54-65%) in farmed European wild boars [7], 20.6% in wild boars in Germany [14], and 9.1% in wild boars in the Czech Republic [5]. The seroprevalence of this study in the wild boar population in the ROK was lower than that (56.4%) in the domestic pig population in ROK [15].

In previous reports, Tomanova *et al.* assumed that wild boars are infected orally, although wild boars may not be exposed to *L. intracellularis* infections from feces to the same extent as domestic pigs kept under intensive farming conditions [4,5]. Regarding the recent publication stating that rodents may be an important reservoir of *L. intracellularis*[16], *L. intracellularis* could be transmitted from domestic pigs to wild animals through infected rodents, and then the infection could be maintained within individual social groups of animals, e.g., within domestic pigs or within domestic pigs, through fecal contamination. However, the possibility that wild boars infected with *L. intracellularis* could be a significant transmission source of this disease to domestic pigs cannot be ruled out. In addition, the infections may persist because wild pigs infected with *L. intracellularis* do not get treated with antibiotics.

The author acknowledges the limitation that seroprevalence only indicates exposure to the agent and it does not indicate ongoing infection nor does it indicate shedding of the bacteria in amounts sufficient to infect other animals. Therefore, the high seroprevalence in wild boars warrants further studies to evaluate their potential as a reservoir species.

Wild boars (*Sus scrofa*) are distributed throughout Asia, Europe, and Northwest Africa, and at least 16 subspecies are currently recognized [17-19]. The Korean wild boar (*Sus scrofa coreanus*) is a common inhabitant of fields and forests on the Korean Peninsula [20]. The results of the current study confirm that *L. intracellularis* is present in the wild boar population worldwide, even in Far East Asia.

## Conclusion

Our results confirm that *L. intracellularis* is present in the wild boar population worldwide, even in Far East Asia. Despite the high seroprevalence shown in wild boars, further studies are warranted to evaluate their potential as a reservoir species because seroprevalence does not prove ongoing infection nor shedding of the bacteria in amounts sufficient to infect other animals. It should also be determined whether the wild boar, like the domestic pig, is a natural host of *L. intracellularis*.

## Methods

### Wild boar serum samples used

Wild boar serum sample collection took place between December 2010 and April 2011 by local governments. Samples were selected from national serum specimen resources originally obtained for the purpose of foot-and-mouth disease surveillance as an emergency response during the 2010/2011 foot-and-mouth disease epidemic [21,22]. Original wild serum samples were collected with the help of hunters, farm workers, or local government officers under the Korea National Animal Health Monitoring Project. Blood from the pericardium or thoracic cavity was taken immediately after killing them or if the wild boars were caught alive, the blood samples for serological analyses were taken by venipuncture from the vena cava cranialis, vena jugularis or the ear vein. Whole blood was collected in 5-ml plastic EDTA tubes, and whole blood for serum was collected in 10-ml plastic blood serum tubes. The tubes were cooled after sampling and sent to the laboratory of the Animal, Plant, and Fisheries Quarantine and Inspection Agency, Anyang, Gyeonggi-do. Blood and serum samples were stored at – 70°C and – 20°C, respectively, until examination. In this study, only serum samples were tested for detection of antibodies against *L. intracellularis* using IPMA*.* All provinces in ROK were included in this study (Table 1). Sample size calculations were performed using the following formula [9,23,24]: *n* = [*Z*_1-a/2_*/d(Se + Sp*-1)]^2^*p*(1-*p*), where *Se* and *Sp* are the sensitivity and specificity of the test, respectively, and *p* is the assumed prevalence. Although we tried to estimate the assumed prevalence from published data on *L. intracellularis* positivity in the wild boar population, the range of prevalence previously reported was wide, from 59.2 to 9.1% [5,7,14]. Because there were no consistent data on the infection frequency when this study was being planned, the necessary sample size was based on an assumed prevalence of *p* = 0.50 to maximize the sample size. A minimal sample size of 208 at the 95% confidence level was required according to the above formula.

### IPMA

Blood samples were examined for the presence of antibodies against *L. intracellularis* by IPMA. Using the pathogenic isolate PHE/KK421 (Korean Collection for Type Cultures 10686BP) [25], IPMA was performed as previously described [26]. Briefly, the acetone-methanol-fixed *L. intracellularis* culture plate was incubated with sera diluted 1:30 in phosphate-buffered saline (PBS) for 30 min at 37°C and washed 5 times with PBS, pH 7.2. Peroxidase-labeled goat anti-porcine IgG was diluted 1:1000 (KPL, MD, USA) in 2% bovine serum albumin and 0.08% Tween 80 in PBS and then added at a concentration of 30 μL/well. The plate was incubated for 45 min at 37°C. The plate was washed again, and a chromogenic (3-amino-9-ethyl-carbazole, Dako Corporation, CA, USA) solution was added to each well. The plate was then incubated at room temperature for 20 min. The plate was washed with distilled water three times, allowed to dry, and examined using an inverted light microscope (Olympus, Tokyo, Japan). *L. intracellularis*-positive and *L. intracellularis*-negative antiserum controls and a secondary antibody control were included on each plate. Positive samples contained red-labeled bacteria, both in the cytoplasm of infected McCoy cells and in the supernatant. Negative control plates using mock-infected cells were included for each individual serum sample to avoid false-positive results.

True prevalence (TP) was estimated, as described by Marchevsky et al. [9-12], using published IPMA test sensitivity and specificity of 100% and 90%, respectively [26]. The formula used to determine TP was: TP = (apparent prevalence + specificity-1)/(sensitivity + specificity-1). Statistical analyses were performed with the NCSS 2007 Statistical Software package (NCSS Statistical System for Windows, Kaysville, UT, USA) and the program ‘Survey Toolbox Version 1.04’ [27].

## Competing interests

The author declares that he has no competing interests.

## Authors’ contributions

JYY conceived of the study, designed the experiments, conducted the majority of the experiments in the laboratory including the cultivation of *L. intracellularis* and McCoy cells and IPMA, wrote the manuscript, conducted analysis of wild boar serum samples and data results, and finalized the manuscript.
